# Why is Real-World Visual Object Recognition Hard?

**DOI:** 10.1371/journal.pcbi.0040027

**Published:** 2008-01-25

**Authors:** Nicolas Pinto, David D Cox, James J DiCarlo

**Affiliations:** 1 McGovern Institute for Brain Research, Massachusetts Institute of Technology, Cambridge, Massachusetts, United States of America; 2 Department of Brain and Cognitive Sciences, Massachusetts Institute of Technology, Cambridge, Massachusetts, United States of America; 3 The Rowland Institute at Harvard, Cambridge, Massachusetts, United States of America; University College London, United Kingdom

## Abstract

Progress in understanding the brain mechanisms underlying vision requires the construction of computational models that not only emulate the brain's anatomy and physiology, but ultimately match its performance on visual tasks. In recent years, “natural” images have become popular in the study of vision and have been used to show apparently impressive progress in building such models. Here, we challenge the use of uncontrolled “natural” images in guiding that progress. In particular, we show that a simple V1-like model—a neuroscientist's “null” model, which should perform poorly at real-world visual object recognition tasks—outperforms state-of-the-art object recognition systems (biologically inspired and otherwise) on a standard, ostensibly natural image recognition test. As a counterpoint, we designed a “simpler” recognition test to better span the real-world variation in object pose, position, and scale, and we show that this test correctly exposes the inadequacy of the V1-like model. Taken together, these results demonstrate that tests based on uncontrolled natural images can be seriously misleading, potentially guiding progress in the wrong direction. Instead, we reexamine what it means for images to be natural and argue for a renewed focus on the core problem of object recognition—real-world image variation.

## Author Summary

The ease with which we recognize visual objects belies the computational difficulty of this feat. At the core of this challenge is image variation—any given object can cast an infinite number of different images onto the retina, depending on the object's position, size, orientation, pose, lighting, etc. Recent computational models have sought to match humans' remarkable visual abilities, and, using large databases of “natural” images, have shown apparently impressive progress. Here we show that caution is warranted. In particular, we found that a very simple neuroscience “toy” model, capable only of extracting trivial regularities from a set of images, is able to outperform most state-of-the-art object recognition systems on a standard “natural” test of object recognition. At the same time, we found that this same toy model is easily defeated by a simple recognition test that we generated to better span the range of image variation observed in the real world. Together these results suggest that current “natural” tests are inadequate for judging success or driving forward progress. In addition to tempering claims of success in the machine vision literature, these results point the way forward and call for renewed focus on image variation as a central challenge in object recognition.

## Introduction

Visual object recognition is an extremely difficult computational problem. The core problem is that each object in the world can cast an infinite number of different 2-D images onto the retina as the object's position, pose, lighting, and background vary relative to the viewer (e.g., [1]). Yet the brain solves this problem effortlessly. Progress in understanding the brain's solution to object recognition requires the construction of artificial recognition systems that ultimately aim to emulate our own visual abilities, often with biological inspiration (e.g., [2–6]). Such computational approaches are critically important because they can provide experimentally testable hypotheses, and because instantiation of a working recognition system represents a particularly effective measure of success in understanding object recognition. However, a major challenge is assessing the recognition performance of such models. Ideally, artificial systems should be able to do what our own visual systems can, but it is unclear how to evaluate progress toward this goal. In practice, this amounts to choosing an image set against which to test performance.

**Figure 1:**
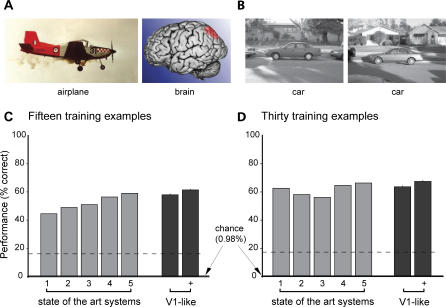
Performance of a Simple V1-Like Model Relative to Current Performance of State-of-the-Art Artificial Object Recognition Systems (Some Biologically Inspired) on an Ostensibly “Natural” Standard Image Database (Caltech101) (A) Example images from the database and their category labels. (B) Two example images from the “car” category. (C) Reported performance of five state-of-the-art computational object recognition systems on this “natural” database are shown in gray (1 = Wang et al. 2006; 2 = Grauman and Darrell 2006; 3 = Mutch and Lowe 2006; 4 = Lazebnik et al. 2006; 5 = Zhang et al. 2006). In this panel, 15 training examples were used to train each system. Since chance performance on this 102-category task is less than 1%, performance values greater than ∼40% have been taken as substantial progress. The performance of the simple V1-like model is shown in black (+ is with “ad hoc” features; see Methods). Although the V1-like model is extremely simple and lacks any explicit invariance-building mechanisms, it performs as well as, or better than, state-of-the-art object recognition systems on the “natural” databases (but see Varma and Ray 2007 for a recent hybrid approach, that pools the above methods to achieve higher performance). (D) Same as (C) except that 30 training examples were used. The dashed lines indicates performance achieved using an untransformed grayscale pixel space representation and a linear SVM classifier (15 training examples: 16.1%, SD 0.4; 30 training examples: 17.3%, SD 0.8). Error bars (barely visible) represent the standard deviation of the mean performance of the V1-like model over ten random training and testing splits of the images. The authors of the state-of-the-art approaches do not consistently report this variation, but when they do they are in the same range (less than 1%). The V1-model also performed favorably with fewer training examples (see Figure S4).

Although controversial ([7,8]), a popular recent approach in the study of vision is the use of “natural” images [7,9–12], in part because they ostensibly capture the essence of problems encountered in the real world. For example, in computational vision, the Caltech101 image set has emerged as a gold standard for testing “natural” object recognition performance [13]. The set consists of a large number of images divided into 101 object categories (e.g., images containing planes, cars, faces, flamingos, etc.; see Figure 1A) plus an additional “background” category (for 102 categories total). While a number of specific concerns have been raised with this set (see [14] for more details), its images are still currently widely used by neuroscientists, both in theoretical (e.g., [2,15]) and experimental (e.g., [16]) contexts. The logic of Caltech101 (and sets like it; e.g., Caltech256 [17]) is that the sheer number of categories and the diversity of those images place a high bar for object recognition systems and require them to solve the computational crux of object recognition. Because there are 102 object categories, chance performance is less than 1% correct. In recent years, several object recognition models (including biologically inspired approaches) have shown what appears to be impressively high performance on this test—better than 60% correct [4,18–21], suggesting that these approaches, while still well below human performance, are at least heading in the right direction.

However, we argue here for caution, as it is not clear to what extent such “natural” image tests actually engage the core problem of object recognition. Specifically, while the Caltech101 set certainly contains a large number of images (9,144 images), variations in object view, position, size, etc., between and within object category are poorly defined and are not varied systematically. Furthermore, image backgrounds strongly covary with object category (see Figure 1B). The majority of images are also “composed” photographs, in that a human decided how the shot should be framed, and thus the placement of objects within the image is not random and the set may not properly reflect the variation found in the real world. Furthermore, if the Caltech101 object recognition task is hard, it is not easy to know what makes it hard—different kinds of variation (view, lighting, exemplar, etc.) are all inextricably mixed together. Such problems are not unique to the Caltech101 set, but also apply to other uncontrolled “natural” image sets (e.g., Pascal VOC [22]).

## Results

To explore this issue, we used the simplest, most obvious starting point for a biologically inspired object recognition system—a “V1-like” model based roughly on the known properties of simple cells of the first primate cortical visual processing stage (area V1). In particular, the model was a population of locally normalized, thresholded Gabor functions spanning a range of orientations and spatial frequencies (see Methods for details). This is a neuroscience “null” model because it is only a first-order description of the early visual system, and one would not expect it to be good for real-world object recognition tasks. Specifically, it contains no explicit mechanisms to enable recognition to tolerate variation in object position, size, or pose, nor does it contain a particularly sophisticated representation of shape. Nevertheless, null models are useful for establishing baselines, and we proceeded to test this null model on a gold-standard “natural” object recognition task (i.e., Caltech101 [13]), using standard, published procedures [21].

We found that this simple V1-like model performed remarkably well on the Caltech101 object recognition task—indeed, it outperformed reported state-of-the-art computational efforts (biologically inspired or not; see Figure 1). Figure 1 shows the cross-validated performance of two versions of this simple model: one where only the model's outputs are fed into a standard linear classifier, and one where some additional ad-hoc features are also used (e.g., local feature intensity histograms; see Methods for details). In both cases, performance is surprisingly good (61% and 67% correct with 15 and 30 training examples), and comparable to, or better than, the current reported performance in the literature ([4,18–21]; but see [23]).

Given the V1-like model's surprisingly good performance on this “natural” image set (Figure 1), there are two possibilities. Either this model is a previously overlooked good model of visual object recognition, or current “natural” tests do not appropriately engage the object recognition problem. Given that our V1-like model contains no special machinery for tolerating image variation (and it would generally be considered a “straw man” model by neuroscientists), we were suspicious that this result had more to do with the test set, than the model itself. Nevertheless, to distinguish between these two possibilities, we designed a second more carefully controlled object recognition test that directly engages the core problem of object recognition.

**Figure 2:**
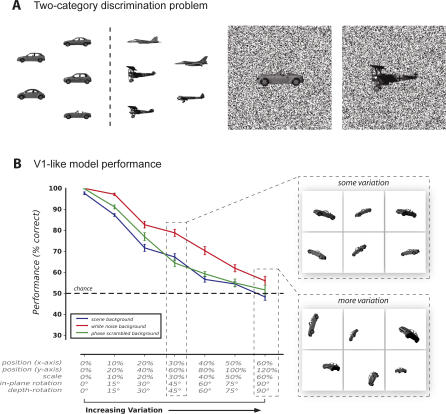
The Same Simple V1-Like Model That Performed Well in Figure 1 Is Not a Good Model of Object Recognition—It Fails Badly on a “Simple” Problem That Explicitly Requires Tolerance to Image Variation (A) We used 3-D models of cars and planes to generate image sets for performing a cars-versus-planes two-category test. By using 3-D models, we were able to parametrically control the amount of identity-preserving variation that the system was required to tolerate to perform the task (i.e., variation in each object's position, scale, pose). The 3-D models were rendered using ray-tracing software (see Methods), and were placed on either a white noise background (shown here), a scene background, or a phase scrambled background (these backgrounds are themselves another form of variation that a recognition system must tolerate; see Figure S1). (B) As the amount of variation was increased (*x*-axis), performance drops off, eventually reaching chance level (50%). Here, we used 100 training and 30 testing images for each object category. However, using substantially more exemplar images (1,530 training, 1,530 testing) yielded only mild performance gains (e.g., 2.7% for the fourth variation level using white noise background), indicating that the failure of this model is not due to under-training. Error bars represent the standard error of the mean computed over ten random splits of training and testing images (see Methods). This result highlights a fundamental problem in the current use of “natural” images to test object recognition models. By the logic of the “natural” Caltech101 test set, this task should be easy, because it has just two object categories, while the Caltech101 test should be hard (102 object categories). However, this V1-like model fails badly with this “easy” set, in spite of high performance on the Caltech101 test set (Figure 1).

Specifically, we constructed a series of two-category image sets, consisting of rendered images of plane and car objects. By the logic of the Caltech101 “natural” image test, this task should be substantially easier—there are only two object categories (rather than 102), and only a handful of specific objects per category (Figure 2A). In these sets, however, we explicitly and parametrically introduced real-world variation in the image that each object produced (see Methods). In spite of the much smaller number of categories that the system was required to identify, the problem proved substantially harder for the V1-like model, exactly as one would expect for an incomplete model of object recognition. Figure 2 shows how performance rapidly degrades toward chance-level as even modest amounts of real-world object image variation are systematically introduced in this simple two-category problem (see Figure S2 for a comparable demonstration with more than two object categories). Given this result, we conclude that the “V1-like” model performed well on the “natural” object recognition test (Figure 1), not because it is a good model of object recognition, but because the “natural” image test is inadequate.

These results (re-)emphasize that object recognition is hard, not because images are natural or “complex,” but because each object can produce a very wide range of retinal images. Although the Caltech101 and other such “natural” sets were useful in that they encouraged the use of common performance tests on which all recognition models should compete, the results presented here show that a different direction is needed to create the content of those tests. This question is not simply an academic concern—great effort is now being expended to test object recognition models against a new, larger image set: the “Caltech256.” However, as with its predecessor, it fails to reflect real-world variation, and our “null” V1 model also performs well above chance (24% accuracy with 15 training examples to discriminate 257 categories), and competitively with early published performance estimates on this new set (see Figure S3).

## Discussion

How should we gauge progress in solving object recognition? First, the results presented here underscore that simple chance performance level is far from a good baseline and that our intuitions about “hard” and “easy” recognition problems are often far from correct. Indeed, it is disconcerting how little variation we needed to introduce to break a model that performs quite well according to current “natural' object recognition tests. Thus, simple “null” models (that are able to exploit regularities in the image database) are needed to objectively judge the difficulty of recognition tasks and to establish a baseline for each such task. The V1-like model presented here provides one possible “null” model, and portable code for building and evaluating it is freely available upon request.

Second, the development of appropriate recognition tests is critical to guiding the development of object recognition models and testing performance of neuronal populations that might support recognition [24]. The construction of such tests is not trivial because the issues cut deeper than simple performance evaluation—this is a question of how we think about the problem of object recognition and why it is hard [1]. Because the number of images in any practical recognition database will be small relative to the dimensionality of the problem domain, test images must be chosen in a manner that properly samples this domain so as to capture the essence of the recognition problem and thus avoid “solutions” that rely on trivial regularities or heuristics.

One approach would be to generate a very large database of “natural” images, like the Caltech sets, but captured in an unbiased way (i.e., with great care taken to avoid the implicit biases that occur in framing a snapshot). Done correctly, this approach has the advantage of directly sampling the true problem domain. However, annotating such an image set is extremely labor-intensive (but see the LabelMe project [25], Peekaboom [26], and the StreetScenes dataset [2,27]). More importantly, a set that truly reflects all real-world variation may be too stringent of an assay to guide improvement in recognition models. That is, if the problem is too hard, it is not easy to construct a reduced version that still engages the core problem of object recognition.

Another approach, an extension of the one taken here, would be to use synthetic images, where ground truth is known by design. Paradoxically, such synthetic image sets may in many ways be more natural than an arbitrary collection of ostensibly “natural” photographs, because, for a fixed number of images, they better span the range of possible image transformations observed in the real world (see also the NORB dataset [28]). The synthetic image approach obviates labor-intensive and error-prone labeling procedures, and can be easily used to isolate performance on different components of the task. Such an approach also has the advantage that it can be parametrically made more difficult as needed (e.g., when a given model has achieved the ability to tolerate a certain amount of variation, a new instantiation of the test set with greater variation can be generated). Given the difficulty of real-world object recognition, this ability to gradually “ratchet” task difficulty, while still engaging the core computational problem, may provide invaluable guidance of computational efforts.

While standardized benchmarks are important for assessing progress, designing benchmarks that properly define what constitutes “progress” is extremely difficult. On one hand, a benchmark that captures too little of the complexity of the real world (no matter how complex it may seem at first glance) invites over-optimization to trivial regularities in the test set (e.g., Caltech101). On the other hand, a benchmark that embraces too much of the “real” problem can be too difficult for any model to gain traction (e.g., the detection challenge in Pascal VOC [22]), giving little insight on which approaches are most promising. This problem is compounded by the fact that there are many more *kinds* of image variation in the real world beyond those used in our simple synthetic test set (e.g., lighting, occlusion, deformation, etc.). At the center of this challenge is the need to clearly define what the problem is, why it is difficult, and what results would constitute success. The path forward will not be easy, but it is time for the field to give this problem much more central attention.

## Methods

### A V1-like recognition system.

Area V1 is the first stage of cortical processing of visual information in the primate and is the gateway of subsequent processing stages. We built a very basic representation inspired by known properties of V1 “simple” cells (a subpopulation of V1 cells). The responses of these cells to visual stimuli are well-described by a spatial linear filter, resembling a Gabor wavelet [29–31], with a nonlinear output function (threshold and saturation) and some local normalization (roughly analogous to “contrast gain control”). Operationally, our V1-like model consisted of the following processing steps.

*Image preparation.* First we converted the input image to grayscale and resized by bicubic interpolation the largest edge to a fixed size (150 pixels for Caltech datasets) while preserving its aspect ratio. The mean was subtracted from the resulting two-dimensional image and we divided it by its standard deviation. The resulting image had zero mean, unit variance, and a size of H × W. Because images have different aspect ratios, H and W vary from image to image.

*Local input divisive normalization*. For each pixel in the input image, we subtracted the mean of the pixel values in a fixed window (3 × 3 pixels, centered on the pixel), and we divided this value by the euclidean norm of the resulting 9-dimensional vector (3 × 3 window) if the norm was greater than 1 (i.e., roughly speaking, the normalization was constrained such that it could reduce responses, but not enhance them).

*Linear filtering with a set of Gabor filters.* We convolved the normalized images with a set of two-dimensional Gabor filters of fixed size (43 × 43 pixels), spanning 16 orientations (equally spaced around the clock) and six spatial frequencies (1/2, 1/3, 1/4, 1/6, 1/11, 1/18 cycles/pixel) with a fixed Gaussian envelope (standard deviation of 9 cycles/pixel in both directions) and fixed phase (0) for a total of N = 96 filters. Each filter had zero-mean and euclidean norm of one. This dimensionality expansion approximates the roughly 100-fold increase in the number of primate V1 neurons relative to the number of retinal ganglion cell axons. To speed this step, the Gabor filters were decomposed via singular value decomposition into a form suitable for use in a separable convolution (this is possible because the Gabor filters are of low rank), and the decomposed filters retained at least 90% of their original variation.

*Thresholding and saturation*. The output of each Gabor filter was passed through a standard output non-linearity—a threshold and response saturation. Specifically, all negative output values were set to 0 and all values greater than 1 were set to 1.

*Local output divisive normalization.* The result of the Gabor filtering was a three-dimensional matrix of size H × W × N where each two-dimensional slice (H × W) is the output of each Gabor filter type. For each filter output, we subtracted the mean of filter outputs in a fixed spatial window (3 × 3 pixels, centered) across all orientations and spatial scales (total of 864 elements). We then divided by the euclidean norm of the values in this window (864 elements), except when the norm was less than 1.

### Comparison to other biologically inspired recognition models.

Some of the other models whose performance is shown in Figure 1 were biologically inspired, and thus also have V1-like stages contained within them, as well as additional machinery intended to allow invariant object recognition (e.g., [2,19]). Thus, it might be surprising that the simple V1-like model presented here outperforms those models. Although detailed comparisons are beyond the scope of this study and tangential to our main point, we note that the V1-like model presented here contains a number of differences from the V1-like portions of these other models (higher dimensionality, larger receptive fields, inclusion of threshold nonlinearities, local normalization, etc.) that probably produce better performance than these models.

### Classification.

To test the utility of our V1-like representation for performing object recognition tasks, we performed a standard cross-validated classification procedure on the high-dimensional output of the model.

*Dimensionality reduction.* To speed computation and improve classification performance, we reduced the dimensionality of the model output prior to classification. The output of V1-like model (above) was a stack of 96 output images, one per Gabor filter type. Because the dimensionality of this stack can be very high (up to 2,160,000 output values per input image depending on its size), standard dimensionality reduction techniques were used to prepare the data for classification. Specifically, each of the 96 output images was low-pass filtered (17 × 17 boxcar) and down-sampled to a smaller size (30 × 30). Thus, regardless of the original input image size, the total dimensionality for classification was always 86,400 (30 × 30 × 96). The data were then sphered (i.e., each filter output was standardized by subtraction of its mean and division by its standard deviation across the training image set; see below), and the dimensionality of the representation was further reduced by principal components analysis (PCA), keeping as many dimensions as there were data points in the training set. For the Caltech101 experiments (e.g., Figure 1), the dimensionality of the final feature vector was 1530 or 3060 (depending on the number of training examples: 15 or 30, respectively).

*Additional “ad hoc” features.* To further explore the utility of this V1-like model, we generated some additional easy-to-obtain features and concatenated these to the final feature vector, prior to PCA dimensionality reduction. These features included: raw grayscale input images (downsampled to 100 × 100 by bicubic interpolation; 10,000 features), and model output histograms for some intermediate stages of the model: pre-normalization (one local histogram per quadrant of the image), post-normalization (full image), and post downsampling (full image)—roughly 30,000 features total. No color information was used in these additional features. Throughout the text, results from the system containing these extra “ad hoc” features are reported separately from those obtained with the system that did not have these extra features. These extra features were added to demonstrate what was possible using additional obvious, “cheap” (but still fair) tricks that improve performance without incurring additional conceptual complexity.

*Training.* Training and test images were carefully separated to ensure proper cross-validation. 15 training example images, and 30 testing example images were drawn from the full image set. Sphering parameters and PCA eigenvectors were computed from the training images (see Dimensionality Reduction, above), and the dimensionality-reduced training data were used to train a linear support vector machine (SVM) using libsvm-2.82 [32]. A standard one-versus-all approach was used to generate the multi-class SVM classifier from the training images.

*Testing protocol.* Following training, absolutely no changes to the representation or classifier were made. Each test image was sphered using parameters determined from the training images, projected through the V1-like model onto the eigenvectors computed from the training images, and the trained SVM was used to report the predicted category of the test image

*Number of training and testing images.* Classifiers were trained using a fixed number of examples (15 and 30 example images; see Figure 1C and 1D). The performance scores reported here are the average of performances obtained from ten random splits of training and testing sets. For testing, 30 images were classified per category, except in categories where there were not enough images available, in which case the maximum number of available images was used (e.g., “inline_skate”, the smallest category, has only 31 examples; when 30 examples were used for training, then only one example was available for testing). Since the Caltech101 sets contains a different number of images for each category, care must be taken to ensure that per-category performance is normalized by the number of test examples considered in each category—otherwise, average performance can be biased toward the performance obtained from categories with larger numbers of images available. This is a particular problem for the Caltech101 set, because some of the largest categories are also empirically the easiest (e.g., cars, airplanes, faces, motorbikes). For the performance values reported in this paper, average performance was computed per category, and then these performances were averaged together to obtain an overall performance value (reported in the text and figures).

*Further controls.* To ensure the validity of our results, we undertook a number of checks to verify that the classification procedure used here was correct. Two different SVM front-ends were used (PyML and libsvm command line tools) and produced identical results. To confirm proper cross-validation, we manually inspected training and test set splits to certify that there were no images in common between the two sets (this control was partially motivated by the fact that an earlier version of the Caltech101 dataset contained duplicates). The classification procedure was also repeated with noise images, and for image sets with category labels scrambled; both tests yielded chance performance, as expected.

### Synthetic dataset generation.

Synthetic images of cars and planes were generated using POV-Ray, a free, high-quality ray tracing software package (http://www.povray.org). 3-D models of cars and planes (purchased from Dosch Design and TurboSquid) were converted to the POV-Ray format. This general approach could be used to generate image sets with arbitrary numbers of different objects, undergoing controlled ranges of variation. For example, in Figure 2 each “pooled variation” level on the *x*-axis shows the maximum deviation of each of five object viewing parameters (zero variation is shown in Figure 2A assuming centering in the image). Given a “pooled variation” level, a set of images was generated by randomly sampling each viewing parameter uniformly within its specified maximum deviation (e.g., +/−30° in plane rotation). Each image in the set was the result of using one such parameter sample to render the view of the object on a given background (see Figure S1). 100% position variation is a full non-overlapping shift of the object's bounding box; 100% scale variation is one octave of change.

While this image set is useful for demonstrating the inadequacy of our V1-like model (in spite of its apparent success at the Caltech101 test), we do not believe it represents any sort of new “standard” against which models of object recognition should be tested. Instead, we believe that the *approach* is more important—identifying the problem, generating sets that span limited regions of the problem space, building models, and then “ratcheting” the problem to a higher difficulty level once the limited version of the problem has been solved.

## Supporting Information

Figure S1 Backgrounds Used Model performance for our “simple” two class image set was assessed with the 3-D models rendered onto three types of backgrounds—white noise, phase-scrambled scene images (∼1/f noise), and intact scene images. Performance with each of these types of background is shown in Figure 2.
(3.8 MB TIF)

Figure S2 Performance Fall-Off for Increasing Numbers of Object Categories Figure 2 shows that relatively modest amounts of image transformation push the performance of our simple V1-like model down to chance. Here we show that this fall-off becomes slightly steeper as more categories-to-be-discriminated are added.
(A) Four categories of objects (cars, planes, boats, and animals) were used to measure performance when 2, 3, or 4 categories are considered.
(B) Average identification performance (“is object category X present or not”) is plotted as a function of view variation and number of object categories to be discriminated. Chance performance is 50% for all three lines, because average one-versus-all performance is shown here, not n-way recognition performance (i.e., “which object is present”).
(1.1 MB TIF)

Figure S3 Performance on the Caltech256 1 = Griffin et al. (2007).
(366 KB TIF)

Figure S4 Performance on the Caltech101 as a Function of the Number of Training Examples, Including Small Numbers of Training Examples Points marked with asterisks are not exact, but were estimated from published plots.
(967 KB TIF)
